# Integrating Network Pharmacology and Experimental Validation to Investigate the Mechanisms of Huazhuojiedu Decoction to Treat Chronic Atrophic Gastritis

**DOI:** 10.1155/2020/2638362

**Published:** 2020-12-07

**Authors:** Xinyu Hao, Yu Liu, Pingping Zhou, Qian Jiang, Zeqi Yang, Miaochan Xu, Shaowei Liu, Shixiong Zhang, Yangang Wang

**Affiliations:** ^1^Hebei University of Chinese Medicine, Shijiazhuang 050200, China; ^2^The First Affiliated Hospital of Hebei University of Chinese Medicine, Shijiazhuang 050011, China

## Abstract

**Background:**

Chronic atrophic gastritis (CAG) is an important stage in the normal gastric mucosa's transformation into gastric cancer. Huazhuojiedu decoction (HZJD), a Chinese herbal preparation, has proven clinically effective to treat CAG. However, few studies have explored the mechanism of HZJD in CAG treatment.

**Purpose:**

This study aimed to shed light on the mechanisms underlying HZJD decoction CAG treatment using a network pharmacology approach and experimental validation.

**Methods:**

The active components of HZJD decoction were obtained from the Traditional Chinese Medicine Systems Pharmacology Database and Analysis Platform. Their targets were predicted through the SwissTargetPrediction database. Disease targets were screened using the GeneCards database. The disease and drug prediction targets were intersected to select the common potential therapeutic targets, which then were input into the Search Tool for the Retrieval of Interacting Genes to build a protein-protein interaction network. The “herb-compound-target-disease” and the “herb-target-pathway” network diagrams were constructed in Cytoscape 3.3.0. Gene Ontology and Kyoto Encyclopedia of Genes and Genomes pathway enrichment analysis of effective targets were performed using the Database for Annotation, Visualization, and Integrated Discovery. Finally, the core targets were preliminarily verified by CAG rat model. The gastric mucosa's histopathological changes were observed via hematoxylin-eosin staining. The expressions of MAPK1, AKT1, TNF, VEGFA, and EGFR were detected by western blot and quantitative real-time reverse transcription-polymerase chain reaction.

**Results:**

A total of 155 nodes, including 20 putative targets of HZJD decoction, were selected as core hubs based on topological importance and were closely associated with the regulation of cell proliferation, apoptotic process, and cancer-related pathways (AKT1, TNF, VEGFA, and EGFR) in CAG. Further animal experiments showed that the expression of AKT1 in CAG rats was significantly increased, which was suppressed by HZJD decoction. TNF and VEGFA expression increased in the model group, but did not change in the HZJD group. MAPK1 and EGFR expression showed no significant differences among control, model, and HZJD groups.

**Conclusion:**

Taken together, the results suggest that the components of HZJD decoction can alleviate and prevent the severity of gastric precancerous lesions via AKT1 inhibition in CAG.

## 1. Introduction

Chronic atrophic gastritis (CAG) is a chronic digestive system disease characterized by the thinning of the mucosal layer and the atrophy and loss of gastric mucosal glands. It is classified as a pregastric cancer state [1]. CAG has a high rate of canceration, posing a serious threat to human health. It has been reported that the prevalence of CAG and intestinal metaplasia (IM) is 16% and 13%, respectively, and increases to 27% in countries with a high gastric cancer incidence [2]. The incidence of gastric cancer in patients with CAG or IM is 0.004–0.3% per person each year, indicating that these patients have a high risk for gastric cancer [3]. Therefore, early identification and treatment of CAG patients allow for early identification of neoplasms and reduction in gastric cancer mortality.

Studies have shown that the histological alteration of CAG may be due to *Helicobacter pylori* infection or may be associated with an autoimmune-mediated reaction directed towards parietal cells or their components [4]. Destruction of parietal cells, either autoimmune-driven or as a consequence of *Helicobacter pylori* infection, can either reduce or abolish acid secretion. Moreover, hypochlorhydria and achlorhydria cause an increase in serum gastrin levels, with an increased risk of hyperplasia, which is considered a precursor lesion of neuroendocrine tumors of the gastric mucosa [5].

Traditional Chinese medicine (TCM) is a comprehensive medicinal system that offers beneficial methods for CAG treatment, with reliable therapeutic efficacy and fewer adverse effects. Studies have shown that TCM can reduce or reverse precancerous gastric lesions and prevent the development of gastric cancer [6–8]. Clinical therapy has found that Huazhuojiedu decoction (HZJD), a Chinese herbal preparation composed of *Capillaris* (*Yinchen, Artemisiae Capillaris Herba*)*, Baical Skullcap Root* (*Huangqin, Radix Scutellariae Baicalensis*)*, Hedyotis Diffusa* (*Baihuasheshecao, Hedyotis Diffusae Herba*)*, Indigowoad Root* (*Banlangen, Radix Isatidis*)*, Lobelia Chinensis Lour* (*Banbianlian, Lobeliae Chinensis Herba*)*, Cablin Patchouli Herb* (*Huoxiang, Herba Pogostemonis*)*, Barbated Skullcup Herb* (*Banzhilian, Herba Scutellariae Barbatae*)*, Sophora Flavescens* (*Kushen, Sophorae Flavescentis Radix*), *Coptis Rhizome* (*Huanglian, Rhizoma Coptidis*)*, Gynostemma Pentaphyllum* (*Thunb*.) *Makino* (*Jiaogulan, Gynostemmae Pentaphylli Herba*), and *Fortune Eupatorium Herb* (*Peilan, Herba Eupatorii*), is an effective prescription for the treatment of CAG. HZJD decoction has been widely used in the clinical setting for many years.

We compared the clinical efficacy, gastroscopic efficacy, and pathological effect of HZJD decoction with Alatan Wuwei Wan for precancerous gastric cancer lesions and found that the effective rate in the HZJD group was higher than that in the Alatan Wuwei Wan group, with a statistically significant difference (*P* < 0.05). HZJD can notably improve the pathological state of conditions such as intestinal metaplasia and dysplasia [9]. In addition, a previous trial in rats with CAG revealed that HZJD decoction reduced CAG's pathological score, with superior results to a group that received vitacoenzyme. The effect may be achieved by downregulating the expression level of TAZ protein and up regulating the expression levels of LATS2 and MST1 in gastric mucosal tissue [10].

Similar to other TCM formulas, HZJD decoction is a multicomponent and multitarget agent that achieves its therapeutic efficacy by regulating molecular networks through active components. However, the comprehensive pathway and targets of HZJD decoction are still not fully understood. Based on the theory of systems biology, network pharmacology involves the construction, data analysis, and mining of biological networks in high-throughput omics and finally carries out a multitarget and multipathway biological system network diagram analysis of a drug's active components. This has proven to be a powerful method to analyze the underlying mechanisms of a TCM formula.

**Figure 1:**
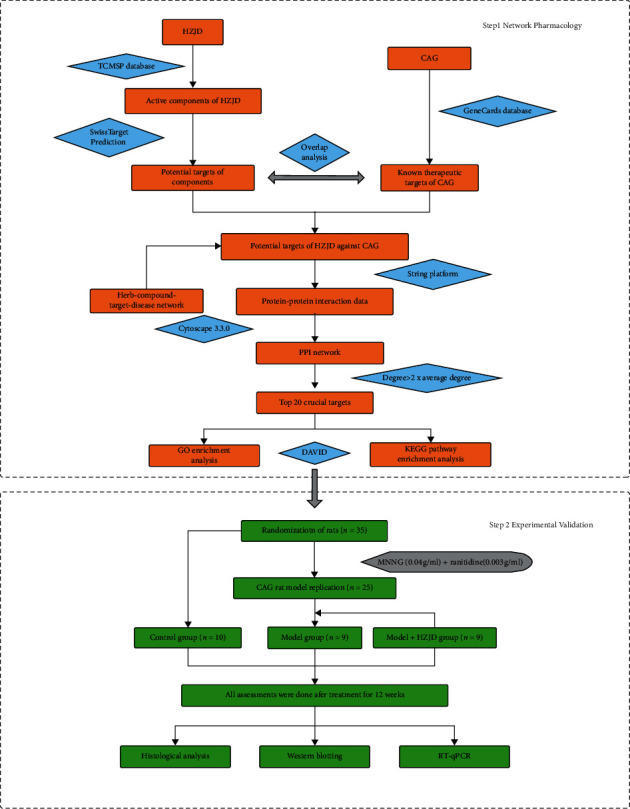
The detailed flowchart of the current study. HZJD, Huazhuojiedu decoction; CAG, chronic atrophic gastritis; TCMSP, Traditional Chinese Medicine Systems Pharmacology; PPI, protein-protein interaction; GO, Gene Ontology; KEGG, Kyoto Encyclopedia of Genes and Genomes.

To further explore and demonstrate the mechanism of HZJD decoction in CAG treatment, this study used a network pharmacology approach to predict HZJD decoction's underlying molecular mechanisms. At the same time, CAG rat models were established to validate the curative role of HZJD decoction as predicted by network pharmacology analysis. The detailed flowchart of the current study is shown in Figure 1.

## 2. Materials and Methods

### 2.1. Network Pharmacologic Analysis

#### 2.1.1. Bioactive Component Screening

All phytochemicals of the 11 constituent herbs of HZJD decoction were retrieved from the TCM Systems Pharmacology Database and Analysis Platform (TCMSP) (http://tcmspw.com/tcmsp.php), a unique systems pharmacology platform of Chinese herbal medicines that captures the associations between drugs, targets, and diseases [11]. The active compounds were screened according to oral bioavailability (OB) and druglikeness (DL). The OB index represents the amount of medication that enters the circulation after oral administration, and the DL index is a qualitative concept used in drug design that estimates how drug-like substance is to be suitable for drug. In this study, values of OB ≥30% and DL ≥0.18 were used as thresholds for screening bioactive components [12].

#### 2.1.2. Obtaining Component Targets

The SwissTargetPrediction (http://www.swisstargetprediction.ch/) database was employed to obtain the targets of active components in HZJD decoction [13]. SwissTargetPrediction is a web server that accurately predicts the targets of bioactive molecules based on a combination of 2D and 3D similarity measures with known ligands. All chemical structures and corresponding canonical simplified molecular-input line-entry systems (SMILES) were retrieved from PubChem (https://pubchem.ncbi.nlm.nih.gov/), an organic small molecule bioactivity database, which is a database of chemical modules [14]. The protein names were standardized through the UniProt database (http://www.uniprot.org/), an informative and resource-rich protein database [15], and the objects were selected as “homo sapiens,” which was the potential target species of the main active components of the drug.

#### 2.1.3. CAG-Related Target Collection

The keyword “chronic atrophic gastritis” was used in the GeneCards database (https://www.genecards.org/) to search for CAG-related targets. GeneCards is a searchable, integrative database that provides comprehensive, user-friendly information on all annotated and predicted human genes [16]. UniProt database was applied to standardize the disease targets. Furthermore, the intersection of the components' targets and CAG disease's targets was determined using the online tool Venny 2.1 (http://bioinfogp.cnb.csic.es/tools/venny/index.html), and the results were taken to be the potential targets for the ingredients of HZJD decoction in CAG treatment.

#### 2.1.4. Construction of Network and Analysis

In order to comprehensively understand the mechanisms of HZJD decoction, the herb-compound-target-disease network was constructed using Cytoscape 3.3.0. In the network, nodes represented herbs, active ingredients, and disease targets. The sides represented the interactions among them. The main network topological parameters were degree and betweenness centrality.

#### 2.1.5. Protein-Protein Interaction Data

The above common target proteins were used to construct the protein-protein interaction (PPI) network model via the Search Tool for the Retrieval of Interacting Genes (STRING) (https://string-db.org/) platform [17], which is a database that searches for protein interactions. Then, the data were imported into Cytoscape for visualization. The topological parameters of nodes in networks were evaluated using cytoscape option network analyzer. A higher degree value node represented a crucial target in the PPI network.

#### 2.1.6. Functional Enrichment and Pathway Analysis

The top 20 proteins according to degree underwent Gene Ontology (GO) and Kyoto Encyclopedia of Genes and Genomes (KEGG) pathway enrichment analysis via the Database for Annotation, Visualization, and Integrated Discovery (DAVID) (https://david.ncifcrf.gov/) [18], which is a database that integrates biological data and analytical tools. GO covers three aspects of biology, including biological processes, molecular functions, and cellular components. KEGG pathway enrichment analysis contributed to the identification of characteristic biological attributes of the potential targets for HZJD decoction in treating CAG [19]. A cutoff value of *P* < 0.05 was used to indicate statistical significance.

### 2.2. Experimental Validation

#### 2.2.1. Animals

All animal experiments were conducted in accordance with the Guide for the Care and Use of Laboratory Animals and were approved by the Animal Care and Use Committee of Hebei University of Chinese Medicine (Shijiazhuang, China). Male Sprague Dawley (SD) rats (250–300 g) were purchased from the Experimental Animal Center of Hebei Medical University (certificate no. 2013-1-003). The animals were housed in a sterile laboratory room, with standard rat chow, at 20–25°C, and in a humidity of 40–70%, with a 12 h light-dark cycle.

#### 2.2.2. Experimental Drugs

HZJD decoction comprises the following components: *Capillaris* (*Yinchen, Artemisiae Capillaris Herba*) 15 g, *Baical Skullcap Root* (*Huangqin, Radix Scutellariae Baicalensis*) 12 g, *Hedyotis Diffusa* (*Baihuasheshecao, Hedyotis Diffusae Herba*) 15 g, *Indigowoad Root* (*Banlangen, Radix Isatidis*) 15 g, *Lobelia Chinensis Lour* (*Banbianlian, Lobeliae Chinensis Herba*) 15 g, *Cablin Patchouli Herb* (*Huoxiang, Herba Pogostemonis*) 9 g, *Barbated Skullcup Herb* (*Banzhilian, Herba Scutellariae Barbatae*) 15 g, *Sophora Flavescens* (*Kushen, Sophorae Flavescentis Radix*) 10 g, *Coptis Rhizome* (*Huanglian, Rhizoma Coptidis*) 12 g, *Gynostemma Pentaphyllum* (*Thunb.*) *Makino* (*Jiaogulan, Gynostemmae Pentaphylli Herba*) 15 g, and *Fortune Eupatorium Herb* (*Peilan, Herba Eupatorii*) 9 g. The herbs were provided and authenticated by Hebei Hospital of Traditional Chinese Medicine. The herbs were boiled with distilled water for 50 min twice and concentrated into a mixture containing 2.8 g/mL of crude drug. N-methyl-N′-nitro-N-nitrosoguanidine (MNNG) was supplied by Tokyo Kabushiki Kaisha, Japan (no. NH8JH-LE). Ranitidine hydrochloride capsules were supplied by Shijiazhuang No. 4 Pharmaceutical Co., Ltd., China (no. LN16050402); Vitacoenzyme capsules were obtained from Dezhou Bocheng Pharmaceutical Co., Ltd., China (no. W20151003).

#### 2.2.3. Reagents and Equipment

Western Lightning^TM^ Chemiluminescence Reagent was obtained from PerkinElmer, USA (lot NEL103 001EA); goat anti-rabbit IgG secondary antibody was supplied by Abcam, UK (lot ab6721); rabbit anti-mouse IgG secondary antibody was supplied by Abcam, UK (lot ab6721ab6728); M-mlv reverse transcriptase was obtained from Takara, Japan (lot 2641A); RNase inhibitors were purchased from Takara, Japan (lot D2310 C); SYBR Premix Ex Taq was obtained from Takara, Japan (lot DRR041 A); Hq-350xt development and fixing equipment was supplied by Suzhou Huqiu Image Equipment Co., Ltd., China; Superrx film was supplied by FUJIFILM, Japan; Decolorizing Orbital Shaker Ts-1 was supplied by Jiangsu Haimen Qilin Bell Instrument Manufacturing Co., Ltd., China; Dycz-24dn vertical electrophoresis device was supplied by Beijing Liuyi Instrument Factory, China; Ve-386 transfer electrophoresis tank was supplied by Beijing Yuanpinghao Biotechnology Co., Ltd., China; 5415D centrifuge was supplied by Eppendorf, Germany; Bio-Rad real-time PCR amplification instrument was supplied by Bio-Rad, USA; MAKT1 antibody was obtained from Cell Signaling Technology, USA (cat.4695S); AKT1 antibody was purchased from Abcam, UK (cat. ab81283); TNF antibody was obtained from Abcam, UK (cat. ab6671); VEGFA antibody was supplied by Abcam, UK (cat.ab1316); and EGFR antibody was purchased from Abcam, UK (cat.ab52894).

#### 2.2.4. CAG Model Establishment and Experimental Protocol

SD rats were randomly divided into three groups: (1) control group, (2) model group, and (3) HZJD group. In accordance with existing literature [20, 21], the CAG rat model was established by treatment with MNNG combined with irregular diet for 20 weeks and with minor modifications. All the model rats were allowed to drink MNNG solution (0.04 g/ml) ad libitum and underwent a hunger-satiety shift every other day. At the same time, the rats were gavaged with ranitidine solution (0.003 g/ml) on the fasting day. At the 12^th^, 16^th^, and 20^th^ weeks, two rats of the model group were randomly sacrificed to perform histological evaluation. According to the histological evaluation, rats of the model group displayed lesions, atrophy, or dysplasia in the 20^th^ week. Rats in the HZJD group were administered HZJD decoction (28 g/kg/day, by gavage) for 12 weeks, while rats in the model group received vehicle (sterile distilled water, by gavage). After treatment for 12 weeks, all rats were sacrificed.

#### 2.2.5. Histological Analysis

The stomach tissue was fixed in 4% buffered paraformaldehyde for 48 h and then dehydrated in alcohol and xylene. Dehydrated samples were embedded in paraffin and cut into sections. The sections were stained with hematoxylin and eosin. The morphological changes were observed under a light microscope and included gastric tissue mucosal layer thickness, glandular morphology and number, epithelial cell arrangement, and infiltration degree of interstitial inflammatory cells.

#### 2.2.6. Western Blotting

Samples were subjected to western blot assay to detect the levels of MAPK1, AKT1, TNF, VEGFA, and EGFR. Total proteins in stomach tissue were lysed for 30 min using RIPA lysis buffer. Lysates were centrifuged at 12,000 rpm for 15 min at 4°C. Then, the protein concentration was measured using a BCA Protein Assay Kit (Thermo Fisher Scientific, USA). Appropriate amount (40 *µ*g) of protein was loaded on 10% sodium dodecyl sulfate-polyacrylamide gel electrophoresis (SDS-PAGE) and transferred to polyvinylidene difluoride (PVDF) membranes. Next, the membranes were sealed with 5% skim milk powder and incubated at 4°C overnight with primary antibodies (MAPK1 1 : 1000 dilution, CST; AKT1 1 : 5000 dilution, Abcam; TNF 1 : 2000 dilution, Abcam; VEGFA 1 : 1000 dilution, Abcam; EGFR 1 : 2000 dilution, Abcam). Subsequently, the membranes were washed with TBST four times for 5 min each time and incubated with secondary antibodies for 1.5 h at room temperature. The membrane was placed into Western Lightning™ Chemiluminescence Reagent (PerkinElmer, USA) colorant for 30 s, and then the photosensitive film was exposed for 1 min in the darkroom. Finally, the bands were visualized by enhanced chemiluminescence. ImageJ software (National Institutes of Health, Bethesda, MD, USA) was used to analyze the intensity of bands.

#### 2.2.7. RT-qPCR

The mRNA levels of MAPK1, AKT1, TNF, VEGFA, and EGFR were determined by the quantitative real-time reverse transcription-polymerase chain reaction (RT-qPCR) method. Total RNA was extracted from stomach tissue using TRIzol reagent (Invitrogen; Thermo Fisher Scientific) according to the manufacturer's instructions. Then, the purity of RNA was evaluated by spectrophotometry (Thermo Fisher Scientific). Subsequently, the RNA was reverse-transcribed into cDNA using the First-Strand cDNA Synthesis kit at 65°C for 10 min, 42°C for 1 h, and 75°C inactivation for 10 min. Next, RT-qPCR was performed using the IQ™5 real-time PCR detection system (Bio-Rad, Hercules, CA, USA) and performed in a reaction mix containing 10 *μ*L Maxima™ SYBR-Green/Fluorescein qPCR Master Mix (2×), 1 *μ*L cDNA, 1 *μ*M forward primer, and 1 *μ*M reverse primer, in a total volume of 20 *μ*L. The cDNA was amplified via primers under the following conditions for 40 cycles: 94°C for 4 min, 94°C for 30 s, and an annealing temperature of 58°C for 30 s, with a final incubation at 72°C for 30 s. The products were analyzed with Ct values, which were normalized against the GADPH RNA level. The final data were analyzed using the 2^−△△Ct^ method. The primer sequences were as follows: MAPK1 forward, 5′-CACTCCATGTAGCTAGAGTGCC-3′ and reverse, 5′-GGAAGACCTGATGGAGACGAC-3′; AKT1 forward, 5′-TGGAGTGTGTGGACAGTGAAC-3′ and reverse, 5′-AGGTACAGATGATCCATGCGG-3′; TNF forward, 5′-GGCTTTCGGAACTCACTGGA-3′ and reverse, 5′-CCCGTAGGGCGATTACAGTC-3′; VEGFA forward, 5′-ACTCATCAGCCAGGGAGTCT-3′ and reverse, 5′-GAGCCCAGAAGTTGGACGAA-3′; EGFR forward, 5′-CCACCAAGACAGGCGACG-3′ and reverse, 5′-AGCAGTAGCTTGGTTCTCGC-3′; and GAPDH forward, 5′-TGGCCTCCAAGGAGTAAGAAAC-3′ and reverse, 5′-GGCCTCTCTCTTGCTCTCAGTATC-3′.

#### 2.2.8. Statistical Analysis

SPSS 21.0 software (SPSS Inc., Chicago, IL, USA) was used to analyze the data. All data were reported as mean ± standard deviation. One-way analysis of variance (ANOVA) was used for multiple-group statistical analyses. The Student–Newman–Keuls (SNK) method was used to analyze the equal variance data. *P* < 0.05 was considered to indicate a statistically significant difference.

## 3. Results

### 3.1. Identification of Potential Bioactive Compounds and Targets in HZJD Decoction

The components of HZJD decoction were retrieved from the TCMSP database. A total of 203 compounds were retrieved from HZJD decoction after eliminating the overlapping compounds. In total, 180 active compounds (Supplementary Table S1) from HZJD decoction were associated with 21,824 target proteins. After eliminating the overlapping proteins, 1249 associated proteins were obtained (Supplementary Table S2).

### 3.2. Candidate Genes Associated with CAG

**Figure 2:**
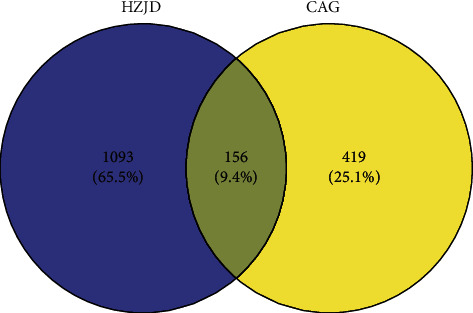
Venn diagram of HZJD decoction and CAG-related targets. The blue part represents the targets of HZJD decoction, the yellow part represents the genes associated with CAG, and the intersection represents the potential targets for the ingredients of HZJD decoction in the treatment of CAG.

A total of 575 significant genes associated with CAG were obtained from the GeneCards database after removing redundant entries (Supplementary Table S3). A Venn diagram was established through Venny 2.1, and 156 genes were obtained as potential targets for the ingredients of HZJD decoction in CAG treatment (Figure 2, Supplementary Table S4).

### 3.3. “Herb-Compound-Target-Disease Network” Construction

**Figure 3:**
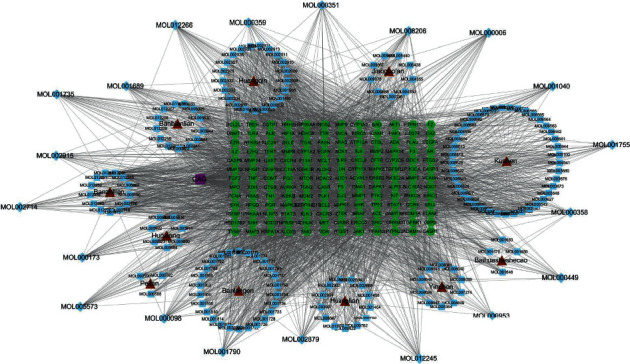
Herb-compound-target-disease network of HZJD decoction acting on CAG. The red nodes represent herbs, the blue rectangle represents the unique components of each herb, the blue diamond represents the common components of each herb, the green nodes represent targets, and the purple nodes represent the disease.

To shed light on the potential mechanisms by which HZJD decoction acts on CAG, 180 active components of HZJD decoction and 156 disease-related target genes were imported into Cytoscape software for network construction and visual display. As shown in Figure 3, the network is composed of 348 nodes (11 constituent herbs of HZJD decoction, 180 bioactive compounds, 156 targets, and the disease). Notably, this network includes some compounds with multiple targets, particularly the high degree and betweenness centrality compounds MOL002911 (2,6,2′,4′-tetrahydroxy-6′-methoxychalcone, degree = 32, betweenness centrality = 0.00588133), MOL001689 (acacetin, degree = 32, betweenness centrality = 0.00270263), MOL012266 (rivularin, degree = 31, betweenness centrality = 0.00290084), MOL001735 (dinatin, degree = 31, betweenness centrality = 0.0023494), MOL002933 (5,7,4′-trihydroxy-8-methoxyflavone, degree = 31, betweenness centrality = 0.00211049), MOL000098 (quercetin, degree = 30, betweenness centrality = 0.00630413), MOL002917 (5,2′,6′-trihydroxy-7,8-dimethoxyflavone, degree = 30, betweenness centrality = 0.00260439), MOL002925 (5,7,2′,6′-tetrahydroxyflavone, degree = 30, betweenness centrality = 0.00260439), and MOL000552 (5,2′-dihydroxy-6,7,8-trimethoxyflavone, degree = 30, betweenness centrality = 0.00244386). These are the important components of HZJD decoction in the treatment of CAG and show statistical significance.

### 3.4. Construction and Analysis of the PPI Network

The obtained key therapeutic targets were input into the STRING database to establish a PPI network and then visualized in Cytoscape (Supplementary Figure 1). As a result, the network was composed of 155 nodes and 2804 edges. In this network, nodes were screened as core hubs if their degree was greater than twofold the average degree value (avg. number of neighbors = 36.181) of all nodes. There were 20 crucial targets, including IL6 (degree = 110), AKT1 (degree = 108), TP53 (degree = 108), ALB (degree = 103), VEGFA (degree = 101), TNF (degree = 96), EGFR (degree = 92), CXCL8 (degree = 91), CASP3 (degree = 89), MAPK3 (degree = 89), STAT3 (degree = 88), SRC (degree = 87), FN1 (degree = 85), MAPK1 (degree = 84), JUN (degree = 83), PTGS2 (degree = 83), MAPK8 (degree = 82), MMP9 (degree = 80), HRAS (degree = 79), and HSP90AA1 (degree = 74).

### 3.5. Functional Enrichment Analysis of Potential Targets of CAG

**Figure 4:**
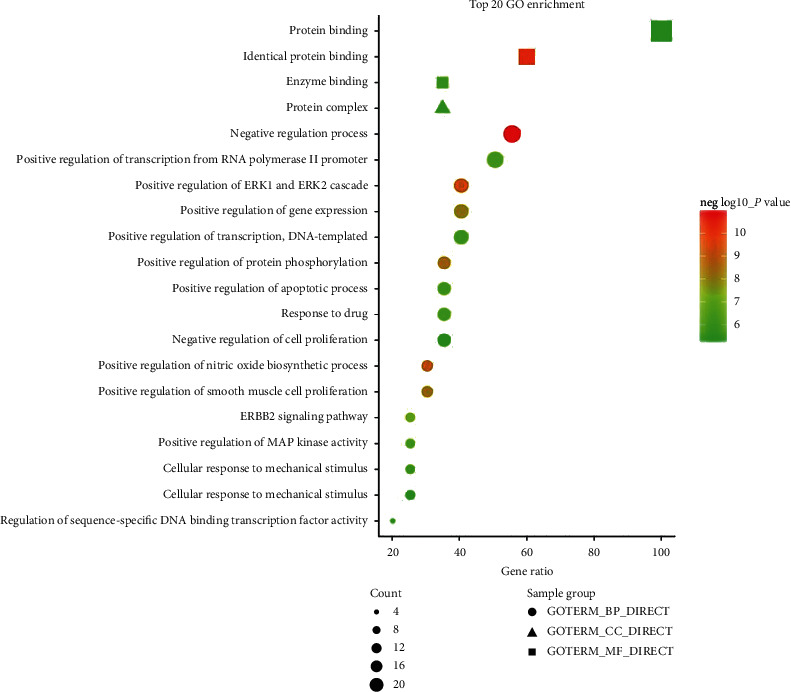
The top 20 results of GO enrichment analysis. Circles represent biological processes, triangles represent cell composition, and squares represent molecular functions. Larger dot size indicates greater enrichment, and as the *P* value decreases, the color becomes increasingly red.

**Figure 5:**
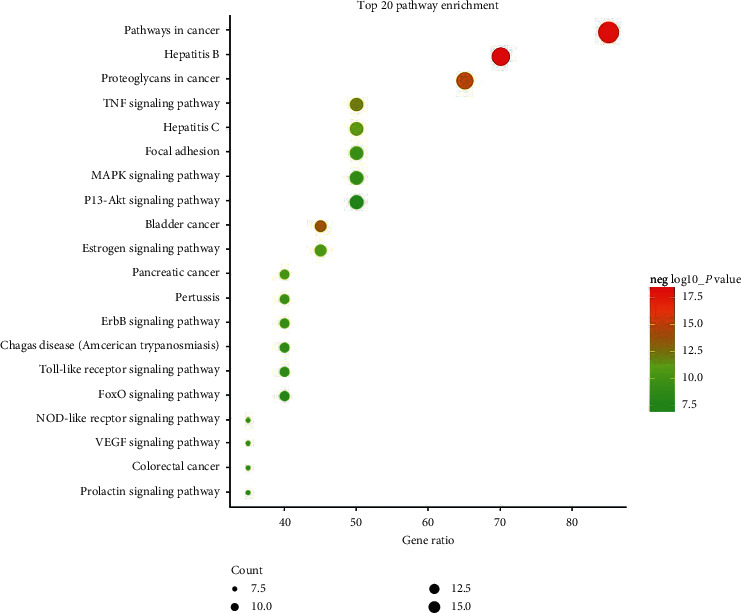
The top 20 pathways of KEGG pathway enrichment analysis. Larger dot size indicates greater enrichment, and as the *P* value decreases, the color becomes increasingly red.

The 20 key targets were input into DAVID for enrichment analysis. To elucidate the multiple biological functions of potential CAG targets from a systematic level, GO enrichment and KEGG pathway enrichment analysis were performed. The top 20 results were selected on the basis of *P* value (Figures 4 and 5).

Also, 10 pathways were selected according to the results of the KEGG pathway enrichment analysis. In addition, the herb-target-pathway network was constructed using Cytoscape to demonstrate relationships (Supplementary Figure 2). The enrichment analysis showed that CAG was closely associated with the regulation of cell proliferation, apoptotic processes, and cancer-related pathways. Thus, the effect of HZJD decoction on the regulation of related targets of rats was observed in the following validation experiment.

### 3.6. Experiment Results

#### 3.6.1. Changes in Gastric Mucosa Morphology

**Figure 6:**
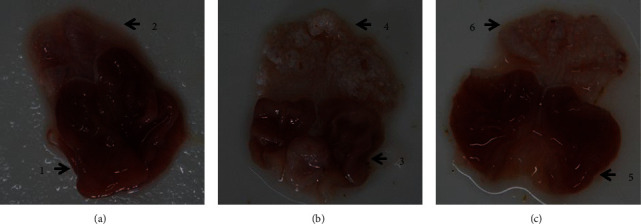
The morphology of gastric mucosa in different groups after HZJD decoction treatment. (a) Control group, (b) model group, and (c) HZJD group

In the normal group, the posterior gastric mucosa was dark red, the mucosa was smooth and shiny, and regular arrangement of mucosal folds could be seen. This region was marked as 1. The anterior gastric mucosa was thin, white, or pale pink, with smooth mucosa and orderly folds, and was marked as 2. In the model group, the posterior gastric mucosa was reddish, the local mucosa was white, and the glossiness was slightly poor. The mucosal plica was low, flat, and decreased, and was marked as 3. The anterior gastric mucosa was thickened and gray-white with a rough surface as well as granular ridges, and the mucosal plica also disappeared. This region was marked as 4. The morphology of the gastric mucosa in the HZJD-treated group was significantly better than the morphology of the model group, which was marked as 5 and 6 (Figure 6).

#### 3.6.2. Histological Analysis

**Figure 7:**
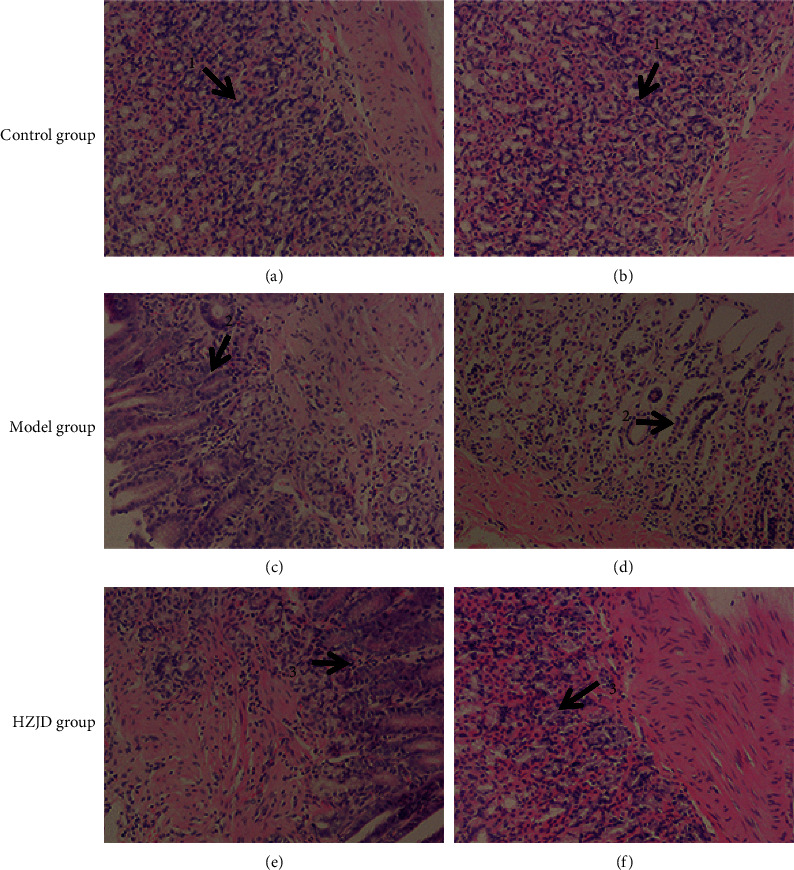
Histological evaluation of CAG in different groups. (a, b) Control group (200×), (c, d) model group (200×), and (e, f) HZJD group (200×).

Histological observation proved to be a powerful tool for evaluating the degree of gastric mucosal lesions. Histologic analysis of the stomachs of different groups after 12 weeks of treatment with HZJD decoction is shown in Figure 7. In the control group, the thickness of the gastric mucosa was normal, the epithelial cells were intact, the glands showed orderly arrangement, and the number was moderate. There was no hyperemia, edema, or inflammatory cell infiltration. The control group's gastric mucosa was marked as 1. In the model group, the gastric mucosa was thinner, the epithelial cells were necrotic and exfoliated, the intrinsic glands were reduced and showed disorderly arrangement, the gastric pits were prolonged, and a large number of inflammatory cells were infiltrated. This was marked as 2. The gastric mucosa of rats in the HZJD decoction group showed the most obvious pathological improvement, with qualities such as moderate mucosal thickness, intact epithelial cells, orderly glandular arrangement, moderate number, and mild hyperemia and edema. The HZJD decoction group's gastric mucosa was marked as 3. As presented in Figure 7, irregular arrangement and inflammatory cell infiltration were observed in the gastric tissues of model group rats compared with those of the control group, which suggested that the CAG rat model was established successfully. Also, the above symptoms were improved after treatment with HZJD decoction, as evidenced by the regular arrangement and marked decrease in inflammatory cell infiltration. These results suggest that HZJD decoction could reduce histological lesions of gastric tissues in CAG rats.

#### 3.6.3. Effect of HZJD Decoction on MAPK1, AKT1, TNF, VEGFA, and EGFR Protein Expressions as Shown by Western Blotting

**Figure 8:**
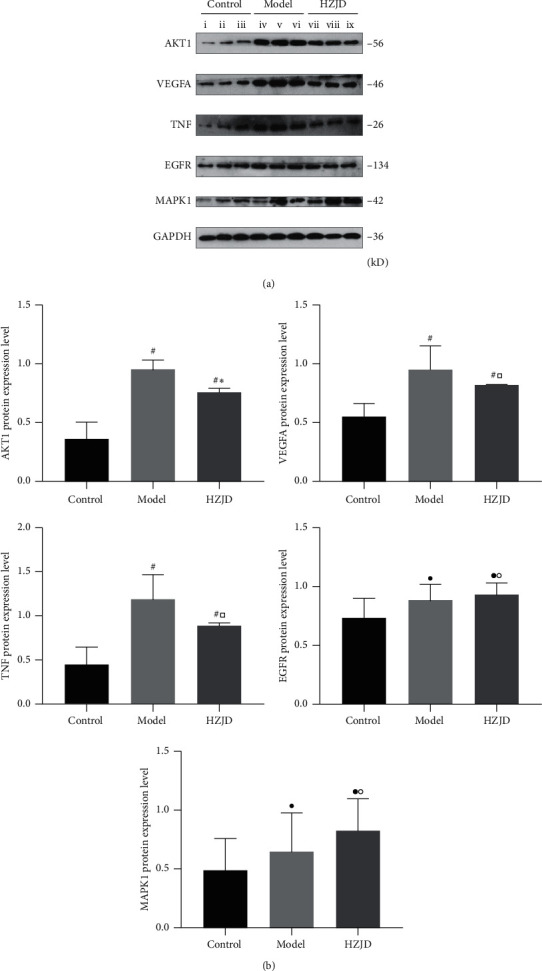
Effect of HZJD decoction on the protein expression of MAPK1, AKT1, TNF, VEGFA, and EGFR in rats. (a) The protein levels were conducted by densitometric analysis of the blots following standardization to GAPDH level; (i, ii, iii) control group, (iv, v, vi) model group, and (vii, viii, ix) HZJD group. (b) Data are presented as the mean ± standard deviation. ^#^*P* < 0.05, ^•^*P* > 0.05, model group and HZJD group vs. control group; ^*∗*^*P* < 0.05, ^□^*P* > 0.05, °*P* > 0.05, model group vs. HZJD group.

Based on the network pharmacology analysis, we selected key proteins associated with signaling pathways, including MAPK1, AKT1, TNF, VEGFA, and EGFR, as the potential drug candidate targets for CAG treatment by HZJD decoction. The protein levels of the above targets were validated by western blotting. As shown in Figure 8, the levels of AKT1, TNF, and VEGFA protein expression in the model group significantly increased when compared with the control group (^#^*P* < 0.05). However, there was no difference in MAPK1 and EGFR expression between the two groups (^•^*P* > 0.05). The 12-week administration of HZJD decoction at both dosages markedly decreased AKT1 expression when compared with that of the model group (^*∗*^*P* < 0.05). However, there was no significant difference in the expression of TNF, VEGFA, MAPK1, and EGFR between the HZJD group and the model group (^□^*P* > 0.05, °*P* > 0.05).

#### 3.6.4. Effect of HZJD Decoction on MAPK1, AKT1, TNF, VEGFA, and EGFR mRNA Levels as Shown by RT-qPCR

**Figure 9:**
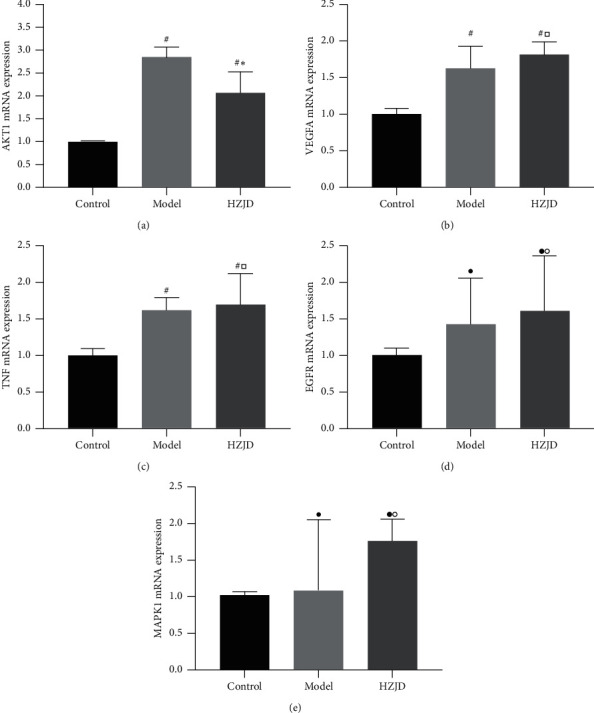
Effect of HZJD decoction on the mRNA expression of MAPK1, AKT1, TNF, VEGFA, and EGFR in rats. Data are presented as the mean ± standard deviation. ^#^*P* < 0.05, ^•^*P* > 0.05, model group and HZJD group vs. control group; ^*∗*^*P* < 0.05, ^□^*P* > 0.05, °*P* > 0.05, model group vs. HZJD group.

The targets were then validated by RT-qPCR (Figure 9). It revealed that CAG rats exhibited notably elevated relative mRNA expressions of AKT1, TNF, and VEGFA compared with the control group (^#^*P* < 0.05). However, the mRNA levels of MAPK1 and EGFR showed no significant change between the two groups (^•^*P* > 0.05). Importantly, HZJD decoction treatment caused a marked drop in AKT1 mRNA levels in CAG tissues (^*∗*^*P* < 0.05). However, it had little effect on TNF, VEGFA, MAPK1, and EGFR (^□^*P* > 0.05, °*P* > 0.05).

## 4. Discussion

Chinese medicine has achieved a remarkable curative effect in the treatment of CAG. As an effective prescription, HZJD decoction has been proven to notably improve pathological changes such as congestion, edema, erosion, and hyperplasia [22]. In addition, it could alleviate many symptoms of CAG, including belching, heartburn, acid reflux, and abdominal distension. HZJD decoction functions in the comprehensive regulation of multiple components and targets. In our study, a total of 180 active compounds from HZJD decoction that were associated with 156 common targets of CAG were obtained. Based on the potential targets of the 180 compounds, network pharmacology analysis was used to obtain 10 associated pathways as well as many associated biological processes, which showed that in theory, HZJD decoction should be highly effective for CAG treatment.

Our analysis determined that some important compounds from HZJD decoction may play roles in CAG treatment. For example, acacetin is a flavonoid compound derived from HZJD decoction component herbs, including *Indigowoad Root* (*Banlangen, Radix Isatidis*), *Lobelia Chinensis Lour* (*Banbianlian, Lobeliae Chinensis Herba*), and *Baical Skullcap Root* (*Huangqin, Radix Scutellariae Baicalensis*). Acacetin, which demonstrates strong antibacterial activity, could inhibit *H. pylori* growth by 86.70% at a dose of only 3.9 lg/mL [23]. It also has been reported that acacetin was able to induce apoptosis in human gastric carcinoma cells [24]. Dinatin, or hispidulin, a naturally occurring flavone from *Indigowoad Root* (*Banlangen, Radix Isatidis*) and *Barbated Skullcup Herb* (*Banzhilian, Herba Scutellariae Barbatae*), can inhibit the growth of gastric cancer cells [25]. Quercetin, a flavonoid, is widely present in HZJD decoction herbs, including *Hedyotis Diffusa* (*Baihuasheshecao, Hedyotis Diffusae Herba*)*, Lobelia Chinensis Lour* (*Banbianlian, Lobeliae Chinensis Herba*)*, Barbated Skullcup Herb* (*Banzhilian, Herba Scutellariae Barbatae*)*, Coptis Rhizome* (*Huanglian, Rhizoma Coptidis*)*, Cablin Patchouli Herb* (*Huoxiang, Herba Pogostemonis*)*, Gynostemma Pentaphyllum* (*Thunb.*) *Makino* (*Jiaogulan, Gynostemmae Pentaphylli Herba*)*, Sophora Flavescens* (*Kushen, Sophorae Flavescentis Radix*), and *Capillaris* (*Yinchen, Artemisiae Capillaris Herba*). According to research, quercetin significantly decreased neutrophil leukocyte infiltration, *H. pylori* colonization, and lipid peroxide concentration in the pyloric antrum when given to *H. pylori*-infected guinea pigs at a dose of 200 mg/kg bw per day [26].

Based on network pharmacology analysis, 20 crucial proteins targeted by these active compounds, including IL6, AKT, TP53, ALB, VEGFA, TNF, EGFR, CXCL8, CASP3, MAPK3, STAT3, SRC, FN1, MAPK1, JUN, PTGS2, MAPK8, MMP9, HRAS, and HSP90AA1, were identified as having potential involvement in CAG treatment. These proteins were primarily associated with the regulation of cell proliferation and apoptotic processes according to the GO enrichment analysis. This is consistent with current research. To the best of our knowledge, numerous studies have proposed that cell proliferation and apoptosis play critical roles in the pathogenesis of CAG [27, 28]. An increase in proliferation and reduction in apoptosis in epithelial cells were the characteristics of atrophic gastritis (AG) and IM. Apoptosis was increased in chronic gastritis and decreased when chronic gastritis progressed to IM or AG [29]. During *H. pylori* infection, the gastric fovea epithelium and glands in the lamina propria are destroyed by direct bacterial toxicity and inflammation. The gastric mucosa may undergo an adaptive repair process leading to intestinal metaplasia and glandular atrophy. Short-term upregulation of apoptosis without a corresponding increase in cell proliferation may lead to cell loss and mucosal damage, and the long-term increase of apoptosis rate may stimulate a continuous increase in cell proliferation and thus promote tumor development [30].

The results of KEGG enrichment analysis indicated that the TNF signaling pathway, ErbB signaling pathway, MAPK signaling pathway, VEGF signaling pathway, and PI3K-Akt signaling pathway were the main pathways underlying CAG treatment by HZJD decoction. Therefore, we established a CAG rat model and selected typical proteins of the associated signaling pathways to verify the curative role and targets of HZJD decoction on CAG as predicted by the network pharmacology analysis. Almost all model rats exhibited severe atrophy. After HZJD decoction administration, severe atrophy lesions were markedly reduced in most CAG rats, which displayed mild and moderate atrophy. In this study, as expected, upregulated expression of AKT1, TNF, and VEGFA was observed in the gastric mucosa of CAG rats compared to expression in normal mucosa. Our results suggested that these proteins may be involved in the malignant transformation. We were also curious as to whether regulation of these proteins was involved in the underlying mechanisms of HZJD treatment of CAG. Interestingly, after HZJD intervention, a decrease in AKT1 levels was frequently concurrent with an improvement in gastric mucosal pathology. Thus, AKT1 may contribute to the activity of HZJD against atrophy. In addition, no significant decrease in TNF and VEGFA levels was observed in HZJD-treated rats, and the expression of MAPK1 and EGFR was found to have no significant differences among control, model, and HZJD groups. This might be due to sample size limitation. It can also be speculated that TNF, VEGFA, MAPK1, and EGFR might not be the potential therapeutic targets by which HZJD treats CAG.

It is known that AKT, the v-AKT murine thymoma viral oncogene homolog, maps to human chromosome 14q32.32 and encodes a 56 kDa protein; it consists of 480 amino acids [31] and plays different roles in cellular processes such as metabolism, proliferation, and apoptosis and is known as the “survival kinase” [32, 33]. AKT is an important effector of the PI3K/AKT/MTOR signaling pathway and has been considered an oncogene essential for tumor initiation and growth [34]. Studies have demonstrated that some AKT haplotypes cause increased AKT protein expression [35]. The genotypes of AKT RS1130233GA and (GA + AA) were associated with an increased risk of atrophic gastritis in *H. pylori*-negative individuals. In addition, p-Akt expression of the rs1130233 mutant of AKT was increased in the *H. pylori-*positive subgroup. Due to the interaction between this polymorphism and *Helicobacter pylori* infection, the effect of this polymorphism on protein expression may only occur during *Helicobacter pylori* infection, which may be the reason why this polymorphism increases the risk of AG [36]. One study's findings implied that the expression of AKT was significantly increased in rats with abnormal glycolysis of MNNG-induced gastric precancerous lesions (GPL), which was a crucial factor in GPL evaluation in terms of glycolysis pathogenesis [37]. Additionally, studies have also shown that *Helicobacter pylori* virulence factors can induce abnormal cell proliferation and apoptosis by regulating signaling pathways (including PI3K/AKT), which lead to gastric cancer [38]. In gastric cancer, the expression of AKT in the cytoplasm is significantly increased in patients with phosphatidylinositol-4, 5-bisphosphonate 3-kinase catalytic subunit (PIK3CA) mutation [39].

For many years, AKT has been considered an attractive target for cancer therapy. Inhibition of the PI3K/AKT pathway prevents uncontrolled cell proliferation. Therefore, the antiapoptotic signal mediated by PI3K/AKT and its downstream molecules has become the focus of drug discovery research in recent years. For instance, antisense oligonucleotides targeting AKT1 have multiple effects on a variety of cancer cell lines, including reducing the ability to grow on soft agar, inducing apoptosis, and increasing sensitivity to various chemotherapeutic agents [40]. Some herbs and natural phytochemicals directly inhibit AKT activity. Oridonin (*Rabdosia rubescens*) inhibits the growth of esophageal squamous cell carcinoma *in vitro* and patient-derived xenografts *in vivo* by targeting AKT [41]. Herbacetin suppresses the growth of squamous cell carcinoma and melanoma cells by targeting AKT and ODC [42]. Panax ginseng C. A. Meyer (Rg3) alleviates gastric precancerous lesions by inhibiting glycolysis through the PI3K/AKT/miRNA-21 pathway [43]. Our study found that HZJD decoction can downregulate the expression level of AKT1 in rats with CAG induced by MNNG. This indicates that targeting AKT to inhibit cell proliferation and promote apoptosis may be the pharmacological mechanism of HZJD decoction in treating CAG. However, this is only a preliminary conclusion, which must be confirmed by further study.

In summary, the active components, targets, and side effects of TCM are an important aspect of TCM modernization studies. Network pharmacology can search for effective substances in complex systems and predict the efficacies of known compounds by constructing networks among genes, proteins, compounds, and toxic reactions, which provide valuable information for understanding their mechanisms of action. However, the network pharmacology approach also has some limitations. First, the retrieval database is not comprehensive enough. Second, some components and targets still lack research support. Finally, the prediction results may be affected by possible biases in the function that are highly studied. Therefore, further experimental verification and other technologies should be used in conjunction with network pharmacology.

## 5. Conclusion

From a network pharmacology perspective and preliminary experimental verification, we can conclude that HZJD decoction can effectively ameliorate the gastric mucosal pathology and inhibit the pathological processes of CAG. Mechanistically, this therapeutic effect may be mediated via the inhibition of AKT1, which may serve as a novel potential target for the clinical treatment of CAG. Although this assumption has been confirmed at the preliminary level, further studies *in vitro* and *in vivo* by gene knockout are also required to assess the direct evidence that AKT1 is associated with the beneficial effect of HZJD decoction on CAG.

## Data Availability

All authors allow researchers to verify the results of an article, replicate the analysis, and conduct secondary analyses.

## Supplementary Materials

Supplementary Materials Table S1: information pertaining to the 180 active compounds of HZJD decoction. Table S2: information pertaining to the 1249 targets of HZJD decoction. Table S3: the 575 significant genes associated with CAG. Table S4: the 156 potential targets for the ingredients of HZJD decoction in CAG treatment. Figure 1: PPI network of 155 common targets. The higher the degree, the redder the color, demonstrating the key targets for the treatment of CAG. Figure 2: herb-target-pathway network of HZJD decoction acting on CAG. The blue nodes represent herbs, the red nodes represent targets, and the gray nodes represent pathways.
