# Supplementary figures and images for: Integrating Network Pharmacology and Experimental Validation to Investigate the Mechanisms of Huazhuojiedu Decoction to Treat Chronic Atrophic Gastritis
Source: Evid Based Complement Alternat Med. 2020 Dec 7;2020:2638362. doi: 10.1155/2020/2638362 (PMC7735863; doi:10.1155/2020/2638362)

## Slide 1
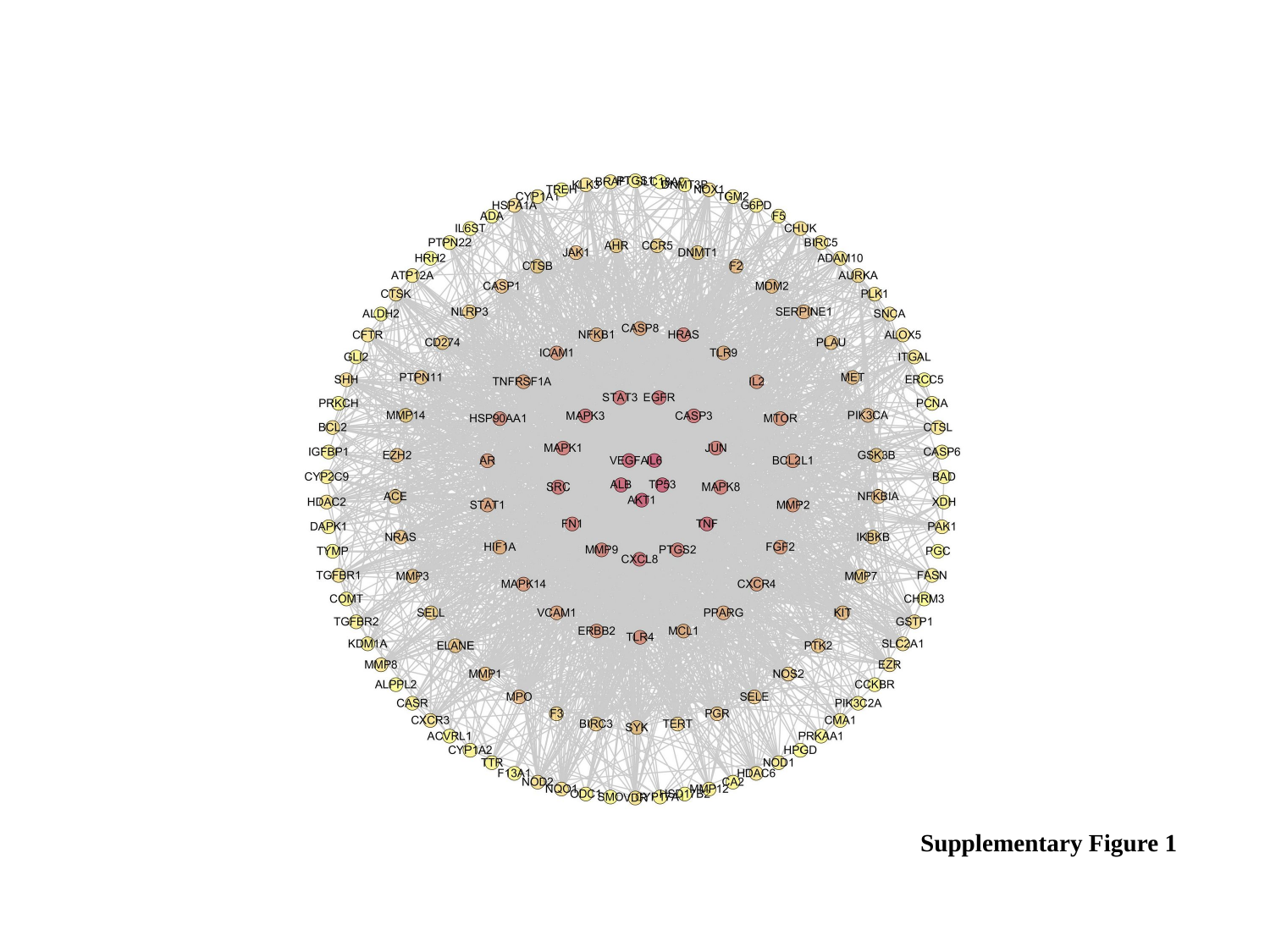

Supplementary Figure 1

Supplement: Supplementary Materials — Table S1: information pertaining to the 180 active compounds of HZJD decoction. Table S2: information pertaining to the 1249 targets of HZJD decoction. Table S3: the 575 significant genes associated with CAG. Table S4: the 156 potential targets for the ingredients of HZJD decoction in CAG treatment. Figure 1: PPI network of 155 common targets. The higher the degree, the redder the color, demonstrating the key targets for the treatment of CAG. Figure 2: herb-target-pathway network of HZJD decoction acting on CAG. The blue nodes represent herbs, the red nodes represent targets, and the gray nodes represent pathways. [file 2638362.f1.zip › Supplementary Materials/Supplementary Figure 1.pptx]

## Slide 1
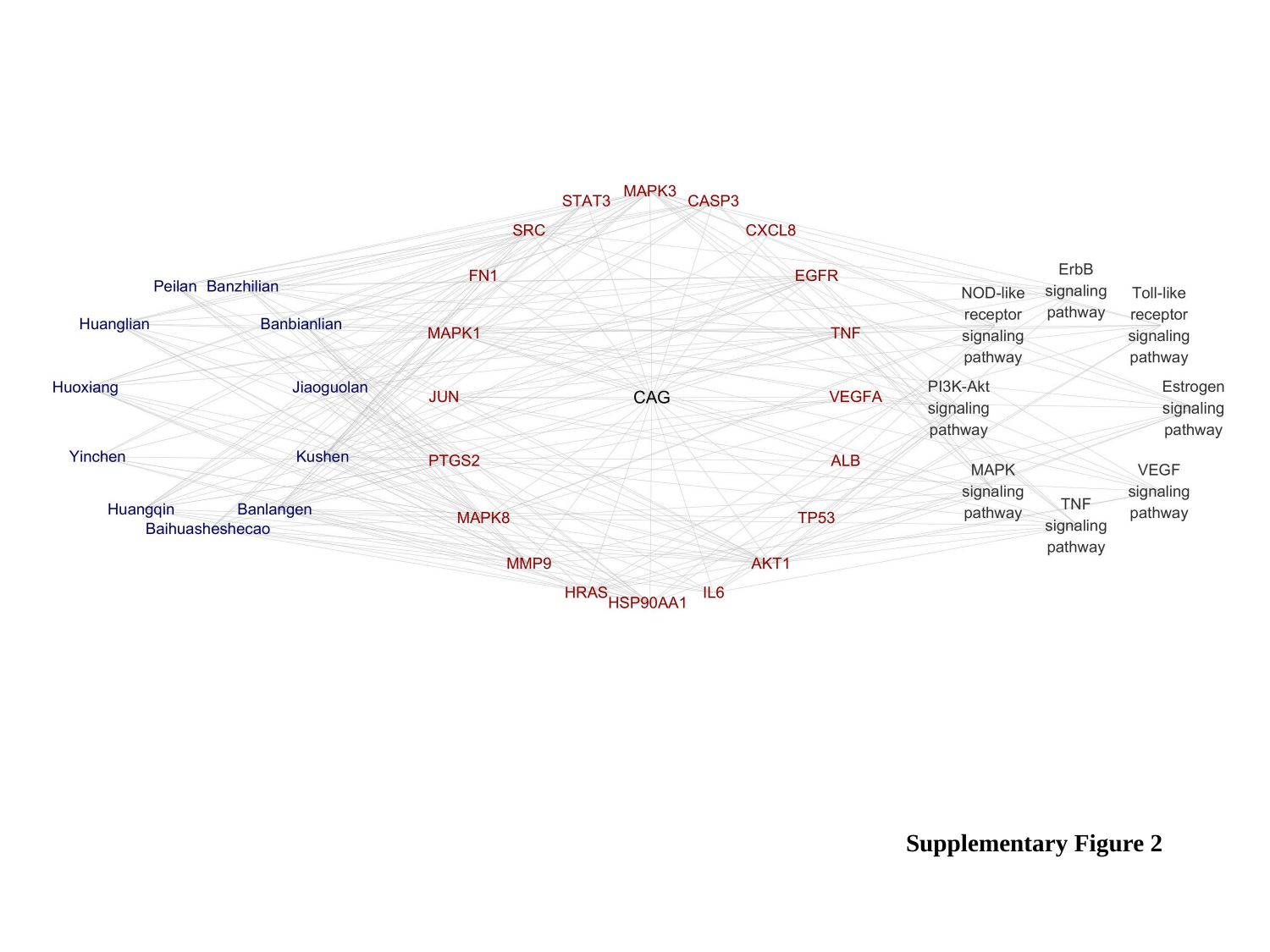

Supplementary Figure 2

Supplement: Supplementary Materials — Table S1: information pertaining to the 180 active compounds of HZJD decoction. Table S2: information pertaining to the 1249 targets of HZJD decoction. Table S3: the 575 significant genes associated with CAG. Table S4: the 156 potential targets for the ingredients of HZJD decoction in CAG treatment. Figure 1: PPI network of 155 common targets. The higher the degree, the redder the color, demonstrating the key targets for the treatment of CAG. Figure 2: herb-target-pathway network of HZJD decoction acting on CAG. The blue nodes represent herbs, the red nodes represent targets, and the gray nodes represent pathways. [file 2638362.f1.zip › Supplementary Materials/Supplementary Figure 2.pptx]
